# Characterization of plasma membrane proteins in *stylosanthes* leaves and roots using simplified enrichment method with a nonionic detergent

**DOI:** 10.3389/fpls.2022.1071225

**Published:** 2022-12-15

**Authors:** Liyun Yang, Jing Gao, Mengze Gao, Lingyan Jiang, Lijuan Luo

**Affiliations:** Hainan Key Laboratory for Sustainable Utilization of Tropical Bioresource, College of Tropical Crops, Hainan University, Haikou, China

**Keywords:** plasma membrane, proteomics, Brij-58 treatment, protein enrichment, *Stylosanthes*

## Abstract

Plant plasma membranes (PMs) play an important role in maintaining the stability of the intracellular environment and exchanging information with the external environment. Therefore, deciphering dynamics of PM proteome provides crucial information for elucidating cellular regulation in response to diverse stimuli. In the study, we developed a simplified method for enriching PM proteins in leaf and root tissues of a tropical forage *Stylosanthes* by combining differential centrifugation and Brij-58 treatment. Both immunoblot analysis and mass spectrometry demonstrated that the representation and abundance of PM proteins were increased in the enrichment fraction, and the contamination of other organellar proteins was decreased. A total of 426 and 388 proteins were predicted to be PM proteins in leaves and roots, respectively. Functional analysis classified these PM proteins into six major categories (transporter, enzyme, receptor, membrane structure protein, vesicular trafficking and chaperone), and orthologs of many PM proteins regulating the responses to abiotic and biotic stresses have been detected. In addition, the sequence analysis, subcellular localization and gene expression analyses of a newly identified receptor-like kinase, SgRKL1, has been performed. Together, these results show that the simplified PM enrichment method can be successfully applied to different plant tissue types and to study the dynamics of PM proteome of *Stylosanthes* in response to multiple stresses.

## 1 Introduction

Due to the difficulties and complexities of whole-cell proteome analyses, subcellular fractionation is an important strategy for focusing on proteins that are of biological interest but present at relatively low levels. About 20-30% of all genes in an organism code for membrane proteins including plasma membrane (PM), and the PM forms the interface between the cytosol and extracellular environments, regulating a broad range of physiological responses, including plant growth and development, sensing and responding to environmental stresses, distribution and movement of biological molecules (ion, hormones, metabolites and etc.) and disease resistance (Chen and Weckwerth, 2020). Due to the unique roles of the PM, identification and functional characterization of plant PM proteome is of great importance.

However, the analyses of PM proteome are challenging due to the high hydrophobicity, heterogeneity and relatively low abundance of PM proteins in the total cellular protein pool (Yadeta et al., 2013; Chen and Weckwerth, 2020). To promote the separation and enrichment of PM proteins, several techniques have been developed, including density gradient centrifugation, free-flow electrophoresis, and phase polymer systems (Yadeta et al., 2013). Aqueous two-phase partitioning is one of the optimal method for isolating highly purified PMs, however, this method is time consuming, and the protocol needs to be optimized for different tissue types, cell types or species (Michele et al., 2016). Because the isolation of subcellular compartments by biochemical approaches can never reach 100% purity, it is of greater interest to develop the strategies that could detect a large number of PM proteins for comparisons rather than isolate highly purified PM proteins. Based on such rationale, a simplified enrichment method using differential centrifugation and Brij-58 treatment has been developed in *Arabidopsis* cell cultures and seedlings as well as maize roots (Zhang and Peck, 2011; Zhang et al., 2013; Collins et al., 2017). Although this method can not produce PM proteins as pure as those from aqueous two-phase partitioning, the strategy is very powerful to enrich sufficient PM fraction for the large-scale identification and quantitative comparisons (Voothuluru et al., 2016).

Stylo (*Stylosanthes* spp.) is a dominant leguminous forage crop cultivated in tropical and subtropical areas (Schultze-Kraft et al., 2018). It is widely used for livestock and soil improvement. Due to its excellent adaptation to acidic and infertile soils, stylo has shown the superior tolerance against phosphorus (P) deficiency, aluminium (Al) and manganese (Mn) toxicity (Sun et al., 2013; Chen et al., 2015; Jiang et al., 2018; Liu et al., 2019; Luo et al., 2020). Interestingly, PM proteins have been shown to play critical roles in regulating these processes. For instance, a transcriptomic analysis that investigated the responses of stylo to Al^3+^ stresses has revealed that the secretion of citrate could be the major mechanisms of Al^3+^ resistance, and anion channels in PM may play vital roles in regulating the secretion (Jiang et al., 2018). In addition, PM localized purple acid phosphatases, SgPAP7, SgPAP10, SgPAP23, SgPAP26, have been proved to mediate the extracellular phytate-P utilization in stylo, contributing to the tolerance of phosphorus deficiency (Liu et al., 2016; Liu et al., 2018). Although roles for several PM proteins of stylo have been identified, broader identification and functional analyses of PM proteins (and their complexes) regulating the resistance under various stresses remain an important area of investigation.

In this study, we evaluated the application of the simplified PM enrichment method of combining differential centrifugation and Brij-58 treatment in the leaf and root tissue of stylo. We demonstrated the easy and effective transfer of the method into new plant species and tissue types. Furthermore, we performed the classification of functions of the detected PM proteins and identified orthologs of many PM proteins with important function. In addition, we performed the sequence analysis, subcellular localization and gene expression analyses of a newly identified receptor-like kinase SgRKL1.

## 2 Materials and methods

### 2.1 Plant growth and tissue collection

The stylo (*Stylosanthes guianensis*) genotype Reyan No.2 seeds (provided by Chinese Academy of Tropical Agricultural Sciences) were used in all experiments. Seeds were peeled and washed by sterilized water, shaken evenly to remove the bubbles and heated at 85°C for 3 min. The heated seeds were germinated on sheets of germination paper moistened with sterilized water at room temperature and near-saturating humidity in the dark. After 3-day germination, seedlings were transferred into soil and grown in 10 cm × 10 cm pots with a density of 10 seedlings per pot. The aerial parts of one-week seedlings were used to collect the leaf tissues. Root tissues were collected from two-week seedlings with primary roots around 20 mm and grown under the hydroponic culture condition where seedlings post 3-day germination were transferred into 1.5 mL eppendorf tubes with culture medium (0.4% plant agar in Magnavaca’s nutrient solution) (Famoso et al., 2010). The temperature for seedling growth ranged from 25°C to 32°C and relative humidity from 60% to 80%.

The inoculation of *Colletotrichum gloeosporioides* was performed as described by previous research (Jiang et al., 2021). Briefly, *C. gloeosporioides* was cultured in CM liquid medium at 200 rpm for 3 days at 28°C, and the mycelium was filtered with sterile gauze to obtain spore suspension. One-month-old stylo plants grown in soil were spray-inoculated of spore suspension with a conidial of 7.5 × 10^6^ conidia/mL containing 0.02% Silwet L-77 (Solarbio, Beijing, China), and the inoculated plants were incubated in dark room at 28°C and 90% humidity for 12 h. Then, the plants were transferred to a growth chamber at 28°C, 90% humidity and 16 h/8 h light/dark photoperiod cycle. Leaf samples pooled from 9 plants were collected at 0 h, 12 h, 24 h, 36 h, 48 h, 60 h, 72 h and 96 h post inoculation. The tissue samples were collected from cotyledons of one-week-old stylo, leaves, stems and roots of one-month-old stylo. For one biological replicate, cotyledon, leaf, stem, root samples were pooled from 9 stylo plants. For low phosphorus (P) stress experiment, stylo seeds were pre-germinated on wet filter paper at 28°C. After germinated for 3 d, the uniform seedlings were transferred to blue plastic basin containing the modified Magnavaca’s nutrient solution according to previous research (Luo et al., 2020). After 15-day growth, the seedlings were transferred to fresh modified Magnavaca’s nutrient solution supplied with (HP) or without (LP) 300 μmol/L KH_2_PO_4_. After 15-day HP and LP treatments, root samples pooled from 9 stylo plants were harvested as one biological replicate. Three biological replicates were included for the following expression pattern analysis.

*Nicotiana benthamiana* plants were grown in a growth room at 22°C with a 16 h/8 h light/dark photoperiod cycle. Four-week-old plants with seven to eight leaves were used for *Agrobacterium tumefaciens* inoculation.

### 2.2 Protein extraction and plasma membrane enrichment

The process of protein extraction and plasma membrane enrichment were modified from the previously published protocol (Collins et al., 2017). Briefly, fresh leaf tissues were ground using 1 mL ice-cold buffer H (250 mM sucrose, 50 mM HEPES-KOH pH 7.5, 5% glycerol, 50 mM NaPP, 1 mM NaMo, 25 mM NaF, 10 mM EDTA, 0.5% PVP, 3 mM DTT, 1 mM PMSF) per 0.2 g, and frozen root tissues were ground to a fine powder with 1 mL buffer H per 0.5 g fresh weight. The homogenate was then centrifuged for 10 min (10000 × *g*, 4°C),and the resulting supernatant was collected and centrifuged in an ultracentrifuge (Thermo Sorvall WX 100) for 30 min (100000 × *g*, 4°C) to pellet the crude microsomal fractions. The pellets were suspended and incubated in 2 μL of buffer H with 0.02% w/v Brij-58 (Sigma-Aldrich, America) per μg of microsomal protein on ice for 30 min, and then ultracentrifuged for 30 min (100000 × *g*, 4°C). The Brij-58 treatment and ultracentrifugation were repeated one more time to obtain the final enriched plasma membrane protein fraction.

### 2.3 Immunoblotting

Proteins samples (20-30 μg) were separated by 10% SDS-PAGE and transferred to PVDP membrane for 2 h at 65 V on ice. The immunoblotting was performed with primary rabbit antibody anti-AHA (H^+^-ATPase plasma membrane marker, Agrisera), anti-AOX1/2 (alternative oxidase isoforms 1/2, mitochondrial inner membrane marker, Agrisera), and anti-β-actin (cytosolic maker, Agrisera) (Slajcherová et al., 2012). Chemiluminescent detection (Pierce) was performed using horseradish peroxidase-linked goat anti-rabbit antibody (Cell Signaling and Technologies). Coomassie Brilliant Blue R-250 staining (0.1% w/v CBB R-250 in 10% v/v acetic acid and 40% v/v methanol) was used after antibody probing, to visualize levels of total protein on the membrane.

### 2.4 SDS-PAGE analysis and tryptic digestion

Total protein and PM fraction were resuspened in sample-loading buffer and heated to 65°C for 7 min. The protein samples were separated by 10% SDS-PAGE, stained with colloidal Coomassie G-250 overnight and destained in distilled water the following day. Each lane was then cut into 3 gel slices with a razor blade. The proteins from gel slices were reduced with 10 mM DTT, alkylated with 55 mM iodoacetamide and digested within the gel using 0.01 μg/μL trypsin in 25 mM NH_4_HCO_3_ buffer at 37°C overnight. After digestion, peptides were eluted twice with 50% ACN. The extracted peptide mixture was dried by lyophilization.

### 2.5 LC-MS/MS analysis

Lyophilized peptides were dissolved in 0.1% FA and 2% ACN for MS analysis. Peptides were first separated through UltiMate 3000 UHPLC (Thermo) and eluted into the nanoelectrospray ion source of a Q-Exactive HF X LC-MS/MS mass spectrometer (Thermo Fisher Scientific, San Jose, CA), which is operating in data-dependent mode. The buffer solutions used for HPLC were 2% ACN, 0.1% FA (buffer A) and 98% ACN, 0.1% FA (buffer B) with a velocity of 300 nL per minute through the following increment: 0-5 min, 5% buffer B; 5-45 min, 5% to 25% buffer B; 45-50 min, 25% to 35% buffer B; 50-52 min, 35% to 80% buffer B; 52-54 min, 80% buffer B; 54-60 min, 5% buffer B. Survey scans covered the range 350-1500 m/z, and the range for MS/MS spectra was 100-1500 m/z. Charge state rejection was enabled for +2 to +6 with a peak detection window of 10 ppm. Dynamic exclusion was enabled with one count for 30 s.

### 2.6 Peptide and protein identification

All MS/MS spectra were analyzed using MASCOT (server version 2.3) (Perkins et al., 1999) searching against Papilionoideaethe Uniprot database (http://www.uniprot.org/, updated on Jan, 2020) which contained 925535 sequences and *Arabidopsis* database (https://www.arabidopsis.org/, updated on Mar 31, 2005). Carbamidomethyl was specified as a fixed modification, and oxidized methionine, deamidated glutamine were specified as a variable modification. The peptide mass tolerance was set as 20 ppm, and the fragment mass tolerance was 0.05 Da. Peptide and protein identifications were assigned by the Percolator (https://www.elastic.co/guide/en/elasticsearch/reference/7.7/percolator.html, updated on July 28, 2014) at FDR ≤ 0.01 (Käll et al., 2007). The relative abundance of identified peptide was calculated based on IBAQ algorithm (Schwanhäusser et al., 2011).

### 2.7 Domain, phylogenetic and motif analysis of RKL1

The coding sequence of SgRKL1 was determined by sequence alignment between the peptide sequence identified from mass spectrometry and *de novo* transcriptome sequence of stylo previously published in our lab (Jiang et al., 2021). The Conserved Domains (https://www.ncbi.nlm.nih.gov/Structure/cdd/wrpsb.cgi) was used for predicting conserved domain of SgRKL1 protein. The DeepTMHMM (https://dtu.biolib.com/DeepTMHMM) was used for predicting signal peptide and transmembrane domain of SgRKL1. A total of 27 homology sequences of RKL1 were downloaded from NCBI, and the phylogenetic tree was constructed using the MEGA v7.0 by neighbour-joining (NJ) method with 500 bootstrap replicates. The MEME (https://meme-suite.org/meme/doc/meme.html) website was used to predict the motif composition of the RKL1 protein. The maximum motif parameter of the gene was 10, the rest of the parameters remained unchanged.

### 2.8 Subcellular localization of SgRKL1

Full length coding sequence of *SgRKL1* gene was amplified from cDNA of stylo leaf using primer pairs (Fw: CAAGGGTCTAGACCCGGGAATGGTGGCCATTAGCATT; Rv: CGATCAATCAGGATCCTAACTCTACAAGATCATGTTGTT) and Phanta Max Master Mix (Vazyme, Nanjing, China). The amplified sequence was gel purified by FastPure Gel DNA Extraction Mini Kit (Vazyme, Nanjing, China) and cloned into the pCAMBIA2300 plasmid by Xhol and SpeI digestion site and ClonExpress Ultra one step cloning kit (Vazyme, Nanjing, China). The expression vector of SgRKL1 was verified by sequencing and introduced into *Agrobacterium tumefaciens* strain GV3101. The sequence of *SgRKL1* was deposited in NCBI data base with accession number OP837387.

*Agrobacterium* cultures harboring pCAMBIA-SgRKL1-GFP/pCAMBIA-GFP and pEG100-PIP2A-mCherry constructs were mixed in a 1:1 ratio to a final OD_600_ of 1.0 in infiltration buffer (10 mM MgCl_2_, 10 mM MES, pH 5.7) that was infiltrated into four-week-old *N. benthamiana* plants. Two days post infiltration, the leaf tissues were collected for detection of GFP and mCherry fluorescence signals at 488 nm and 587 nm using confocal microscopy (A1RHD25+ N-SIM +N-STORM, Nikon) and protein isolation. Different fractions of leaf of *N. benthamiana* were isolated using the same method as stylo described in section 2.2.

### 2.9 Quantitative real-time PCR analysis

Total RNA was extracted from leaf and root tissues of stylo using TRizol reagent (Invitrogen, Waltham, MA, United States) according to the manufacturer’s protocol. First-strand cDNA was synthesized from 1 μg of total RNA using HiScript II 1st Strand cDNA Synthesis Kit (+gDNA wiper) (Vazyme, Nanjing, China). Quantitative real-time PCR (qRT-PCR) was carried out using ChamQ Universal SYBR qPCR Master Mix (Vazyme, Nanjing, China) on Applied Biosystems QuantStudio™ 7 Flex System (Applied Biosystems, USA). Reaction mixtures of 10 μL contained 5 μL of 2× ChamQ Universal SYBR qPCR Master Mix, 0.2 μL of each primer (0.2 μmol/L, Fw: CCTTCCGGCGACATTACCAA; Rv: GCAGAAGACACACCACGGAT), 1 μL of cDNA template, and 3.6 μL of ddH_2_O. The amplification program was set at 95°C for 3 min, followed by 40 cycles of 95°C for 15 s and 60°C for 1 min, and a melt-curve program (60–95°C, with a temperature increase of 0.05°C after every 1 s). Signal threshold levels were set automatically by the system. The housekeeping gene, *ribosomal protein L19 (RPL19)*, was used as reference gene to normalize gene expression (Jiang et al., 2021). Three biological replicates were included in this study, and the relative expression levels calculated using 2^-ΔΔCt^ (Schmittgen and Livak, 2008).

### 2.10 Statistical analysis

The experimental data was analyzed using SPSS software (Version 20; SPSS Inc., Chicago, IL, USA) with the ANOVA procedures, the relative expression level was displayed with mean ± standard error (SE). Dancan’s method was used for multiple comparison, and Student’s t-test was used for comparison between two groups, *P <*0.05 was considered statistically significant.

## 3 Results

### 3.1 Development of PM-enrichment method in *Stylosanthes* leaves and roots

#### 3.1.1 Outline of procedures

**Figure 1:**
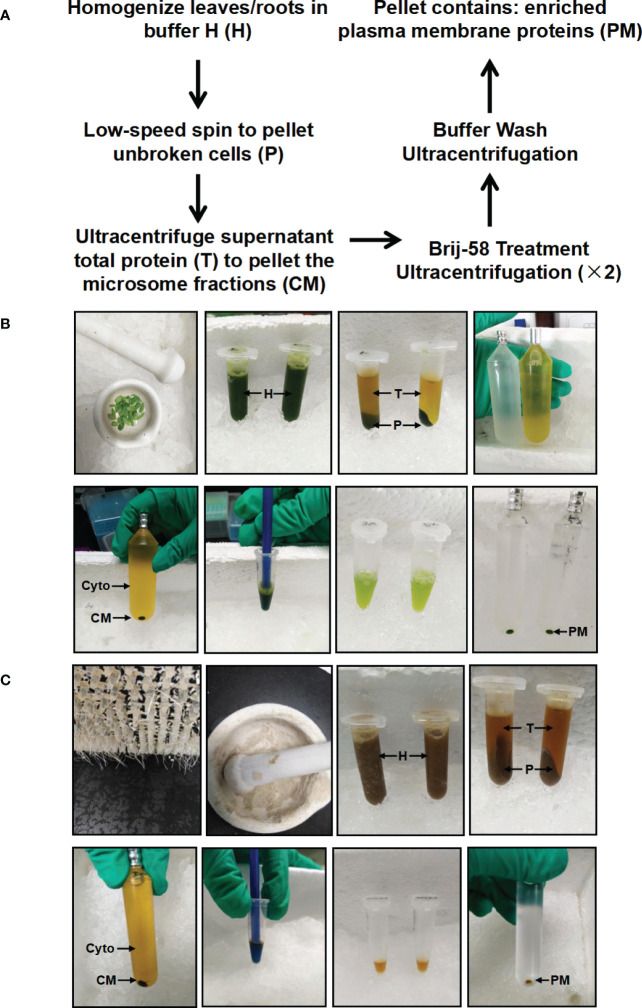
Schematic and image representation of the simplified plasma membrane enrichment procedure in leaf and root tissue of *Stylosanthes*. **(A)** Schematic representation of the procedure. **(B)** Images showing the key procedures in leaf tissue. **(C)** Images showing the key procedures in root tissue. After homogenization in Buffer H, the total homogenate (H) is subjected to low-speed spin to separate the supernatant (T: containing cytosolic and microsomal proteins) from the pellet (P: containing cell debris). The total protein fraction (T) is transferred to ultracentrifugation tube to pellet the crude microsome (CM) by ultracentrifugation, and the resulting supernatant contains cytosolic proteins (Cyto). The CM is resuspended in Buffer H with Brij-58, incubated on ice and subjected to ultracentrifugation to yield an enriched plasma membrane fraction (PM).

In this study, we have developed a simplified method to enrich PM proteins in stylo leaf and root tissues using differential centrifugation and Brij-58 treatment (**Figure 1**). Briefly, frozen leaf or root tissues were ground with a motar and pestle. Following homogenization, protein extracts were pre-cleared by a low-speed centrifugation to remove cell debris and intact organelles. The resulting supernatant total protein (T) was used to pellet the crude microsomal fraction (CM) by ultracentrifugation. The CM fraction was resuspended in buffer H containing Brij-58, and the suspension was pelleted by ultracentrifugation followed by buffer wash to remove loosely bound membrane-associated proteins. After the final round of ultracentrifugation, the resulting pellet was hereafter referred to as the enriched plasma membrane fraction (PM) (**Figure 1**).

#### 3.1.2 Immunoblot analysis of different fractions

**Figure 2:**
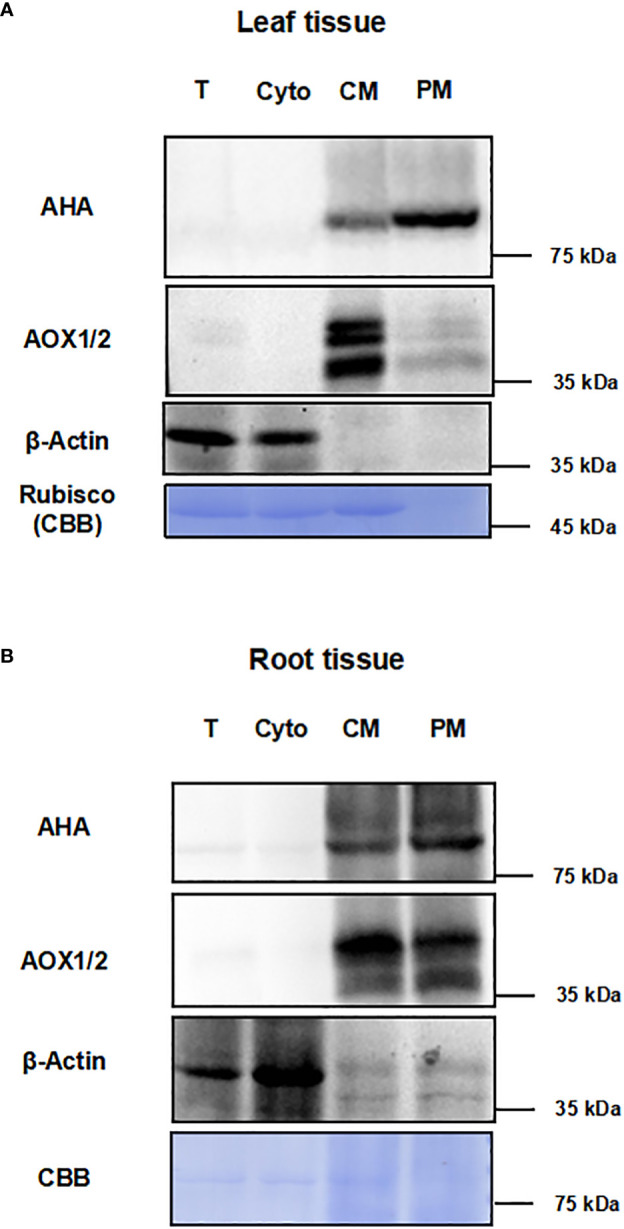
Immunoblot analysis for different fractions with antibodies against subcellular marker proteins. The proteins of leaf and root tissue from steps of the enrichment procedure were analyzed by immunoblotting with organelle specific markers (T, total homogenate; Cyto, cytosolic fraction; CM, crude microsomal fraction; PM, plasma membrane-enriched fraction). **(A)** Equal amounts of proteins of leaves from each fraction were analyzed by immunoblotting with two antibodies against organellar marker proteins (PM marker: AHA; mitochondrial marker: AOX1/2; cytosolic maker: β-actin). **(B)** Equal amounts of proteins of roots from each fraction were analyzed by immunoblotting with antibodies against AHA, AOX1/2 and β-actin. PM, plasma membrane; AHA, H+-ATPase; AOX1/2, Plant alternative oxidase 1 and 2. Total protein loads were detected by coomassie blue staining (CBB).

To test the effect of this method for enriching PM proteins, we performed the immunoblot analysis using equal amounts of protein from total protein fraction (T), the supernatant fraction of first ultracentrifugation (Cyto), the CM fraction and the PM fraction (**Figures 2A, B**). The integral PM H^+^-ATPases (AHAs), mitochondrial inner membrane protein (AOX1/2), β-actin and cytosolic rubisco proteins were used as markers (Slajcherová et al., 2012; Evans and Clarke, 2019). For leaf tissues, the AHA proteins were strongly enriched in the PM as compared to the T, Cyto or CM fraction, and the AOX1/2 proteins were enriched in the CM fraction and depleted in the PM fraction. Conversely, cytosolic markers, β-actin and rubisco proteins, were strongly depleted in PM fraction (**Figure 2A**). For root tissues, the AHAs and AOX1/2 proteins were enriched in the CM and PM faction, and the AOX1/2 proteins were decreased in the PM fraction. The β-actin proteins were depleted in the PM fraction (**Figure 2B**). These results demonstrate that, at least for the markers used, the strategy combining differential centrifugation and Brij-58 treatment has effectively enriched the plasma membrane proteins and reduced the proteins from cytosol and other membrane organelles.

#### 3.1.3 Analysis of total and PM faction by MS

**Figure 3:**
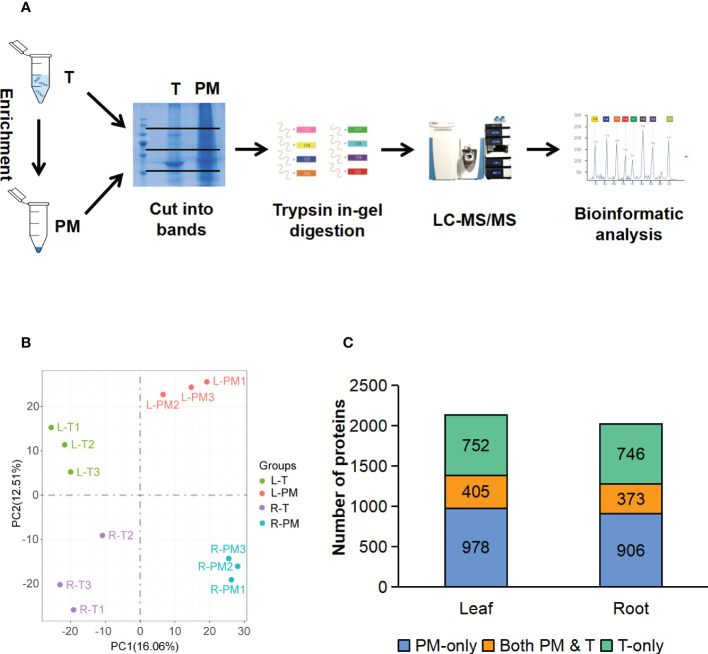
LC-MS/MS analysis of the total fraction and enriched plasma membrane fraction. **(A)** Workflow of MS analysis of total and enriched PM fraction. **(B)** PCA analysis of proteome data from the total and enriched PM fraction of leaf and root tissue, three biological replicates for each fraction of each tissue type. **(C)** Comparison of proteins identified in total fraction alone (green blocks), enriched PM fraction alone (blue blocks), and shared by both fraction (orange blocks). T, total fraction; PM, enriched plasma membrane fraction; L-T, total fraction of leaves; L-PM, enriched plasma membrane fraction of leaves; R-T, total fraction of roots; R-PM, enriched plasma membrane fraction of roots.

To further test the enrichment effect, we applied mass spectrometry (MS) to look at a wider range of proteins. For both leaves and roots, proteins from the total fraction and PM fraction were separated by SDS-PAGE. Each lane was cut into three slices, and peptides were recovered by in-gel digestion, the proteins in the gels were reduced, alkylated, and digested with trypsin. The digested proteins were analyzed by liquid chromatography tandem mass spectrometry (LC-MS/MS) (**Figure 3A**), and all the identified proteins and peptides were summarized in the **Table S1**. The identified proteins were further filtered based on minimum of two unique peptides, resulting in 2135 and 2025 proteins in leaves and roots, respectively (**Table S2**). The PCA analysis of T and PM proteome in leaves and roots showed the clear separation between enrichment treatment (16.06%) and different organs (12.51%) (**Figure 3B**). In leaf tissues, 752 (35%) and 978 (46%) unique proteins were identified in T and PM fraction, respectively, and 405 (19%) proteins overlapped between the T and PM fraction. In root tissues, 746 (37%) and 906 (45%) unique proteins were identified in T and PM fraction, respectively, and 373 (18%) proteins overlapped between the T and PM fraction (**Figure 3C**).

To examine the consistency of the mass spectrometry and immunoblotting results for PM enrichment, we checked the iBAQ values of several representative proteins, of which the localization has been previously published (**Tables 1**, **2**). The results showed that the iBAQ value of the PM marker (AHA) was significantly increased in the PM fraction both in leaves and roots. Similarly, the amount of other classic PM proteins such as aquaporin, LRR receptor-like kinase and calcium-transporting ATPase was also significantly increased after enrichment. Conversely, the representative proteins in MI, ER, Cyto were almost depleted after enrichment. These results clearly demonstrate the ability of this method to enrich PM proteins in both leaves and roots of stylo, we next performed a more extensive analysis to identify the organellar composition in the T and PM fraction.

**Table 1:** Representative list of PM, ER, MI and Cyto proteins in PM and T fractions of leaves measured by mass spectrometry.

Accessionnumber ^(a)^	Arabidopsis ID ^(b)^	Description	iBAQ (Leaf)^(c)^		Compartment^(d)^
PM	T
A0A445C0K6	AT2G24520	Plasma membrane ATPase	860954.80	11790.07	PM
A0A371HFL0	AT3G16240	Aquaporin TIP2-1	64291040.66	0	PM
A0A2K3PLU7	AT5G10290	LRR receptor-like kinase resistance protein	143487.49	0	PM
A0A444WPI3	AT5G49360	Fn3_like domain-containing protein	0	1829698.35	ER
A0A445KRZ9	AT5G42020	Luminal-binding protein 4 isoform D	0	29890.76	ER
A0A371IGU2	AT4G38630	26S proteasome non-ATPase regulatory subunit 4-like protein	10990.20	387695.32	ER
A0A1S2XXJ3	AT3G23990	chaperonin CPN60-2	0	120063.77	MI
A0A444ZVK9	AT4G37930	Serine hydroxymethyltransferase	0	300434.54	MI
A0A445DTY1	AT1G63940	Pyr_redox_2 domain-containing protein	0	314465.04	MI
A0A445E0V9	AT1G05180	NEDD8-activating enzyme E1 regulatory subunit	0	314501.02	Cyto
A0A4D6N6X8	AT5G38830	Cysteinyl-tRNA synthetase	0	497946.91	Cyto
A0A392S5X8	AT1G65350	Ubiquitin 11	0	1575497.93	Cyto

(a) Database accession number from UniProt; (b) Database Arabidopsis ID from Tair; (c) The iBAQ value is the average of three replicates; (d) Compartment assignment based on publication. PM, plasma membrane; ER, endoplasmic reticulum; MI, mitochondria; Cyto, cytosol.

**Table 2:** Representative list of PM, ER, MI and Cyto proteins in PM and T fractions of roots measured by mass spectrometry.

Accessionnumber ^(a)^	Arabidopsis ID ^(b)^	Description	iBAQ (Root)^(c)^		Compartment^(d)^
PM	T
A0A444ZI65	AT2G24520	Plasma membrane ATPase	681078.44	0	PM
A0A445GUH7	AT5G57110	Calcium-transporting ATPase	168601.27	0	PM
A0A445DU07	AT4G11220	Reticulon-like protein	3590495.91	0	PM
A0A151SQ13	AT1G77510	Protein disulfide-isomerase	0	265827.62	ER
A0A445KTK0	AT2G32730	26S proteasome non-ATPase regulatory subunit 1 homolog	76757.07	191063.76	ER
A0A0L9V3F1	AT1G53240	Malate dehydrogenase	0	108372.81	MI
A0A445CGM8	AT5G26780	Serine hydroxymethyltransferase	38394.02	647188.72	MI
A0A444ZMJ2	AT3G61440	Cysteine synthase	0	212915.78	MI
A0A445CYF4	AT3G24170	Glutathione reductase	0	216379.56	Cyto
A0A445DN91	AT2G44100	Guanosine nucleotide diphosphate dissociation inhibitor	0	399519.35	Cyto

(a) Database accession number from UniProt; (b) Database Arabidopsis ID from Tair; (c) The iBAQ value is the average of three replicates; (d) Compartment assignment based on publication. PM, plasma membrane; ER, endoplasmic reticulum; MI, mitochondria; Cyto, cytosol.

### 3.2 Analysis of organellar composition of total versus PM fractions

Protein locations were assigned based on WoLF PSORT (Horton et al., 2007), and proteins with multiple location assignments were excluded from our lists. We first examined T and PM fraction for the percentage of proteins predicted to be in different locations (**Table 3**; **Table S2**). In leaf tissues, we found that the proteins with increased representation in PM fraction were predicted to be localized in the plasma membrane (PM, 2.08-fold enrichment), vacuole (VU, 1.22-fold enrichment), golgis apparatus (GO, 1.26-fold enrichment) and mitochondria (MI, 1.18-fold enrichment) while proteins with decreased number were from cytosol (CY), plastid (PL), extracellular space (EX), peroxisome (PR), cytoskeleton (CS). Proteins from nucleus (NU) and endoplasmic reticulum (ER) were distributed relatively evenly between two fractions. In root tissues, the PM enriched for proteins in the PM (2.00-fold enrichment) and VU (1.18-fold enrichment) while decreased the number of proteins from GO, ER, CY, PL, EX, PR and CS. Proteins from MI and NU were distributed relatively evenly between two fractions (**Table 3**). The results showed that this method has a similar effect of enrichment in leaves and roots, and the representation of PM proteins was increased by more than 2-fold in PM fraction.

**Table 3:** Comparison of organelle proteomes between total protein and enrichment fraction.

Cellular compartments	Leaf			Root		
	% of proteins identified in PM fraction	% of proteins identified in T fraction	Fold change of protein representation (PM/T)	% of proteins identified in PM fraction	% of proteins identified in T fraction	Fold change of protein representation (PM/T)
PM	26.53%	12.75%	2.08	24.65%	12.34%	2.00
VU	2.11%	1.73%	1.22	1.81%	1.53%	1.18
MI	5.23%	4.42%	1.18	5.35%	5.23%	1.02
NU	7.99%	7.29%	1.10	8.82%	7.93%	1.11
GO	0.22%	0.17%	1.26	0.08%	0.09%	0.87
ER	1.24%	1.21%	1.02	1.10%	1.44%	0.76
CY	24.78%	31.22%	0.79	29.37%	34.41%	0.85
PL	28.71%	36.77%	0.78	26.46%	32.25%	0.82
EX	1.31%	1.73%	0.75	0.94%	1.80%	0.52
PR	0.58%	0.78%	0.74	0.24%	0.63%	0.37
CS	1.31%	1.91%	0.69	1.18%	2.34%	0.50

The ratio of the total number of proteins in each subcellular compartment to the total number of proteins identified. The average of three biological replicates is mean percentage of exclusive proteins identified for each cellular compartment. PM, plasma membrane; VU, vacuole; MI, mitochondria; NU, nucleus; GO, golgis apparatus; ER, endoplasmic reticulum; CY, cytosol; PL, plastid; EX, extracellular space; PR, peroxisome; CS, cytoskeleton.

**Figure 4:**
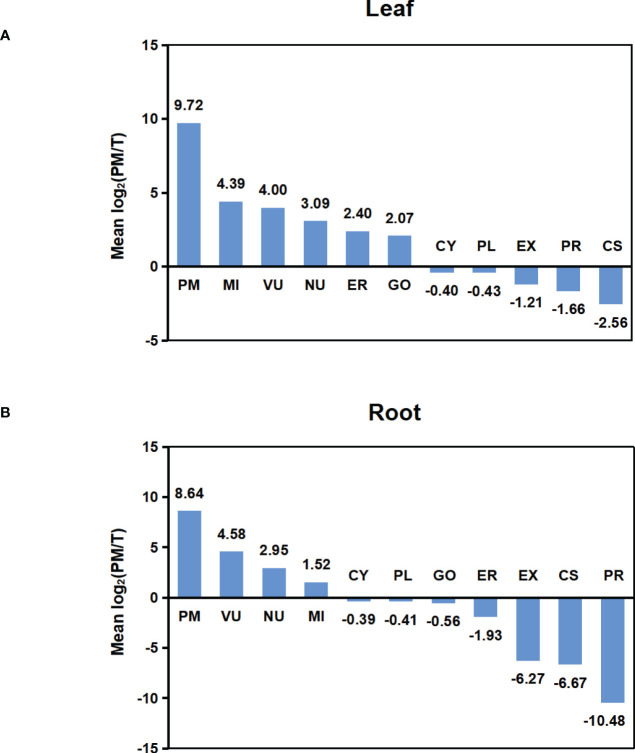
Abundance comparison of organelle proteomes between total and PM enriched fraction. **(A)** Abundance comparison of organelle proteomes between two fractions in leaf. **(B)** Abundance comparison of organelle proteomes between two fractions in root. Log2 values were calculated for the ratio of IBAQ value of proteins assigned to specific compartments in PM or total fraction [i.e. log2(PM/T)]. The mean log2(PM/T) was calculated by using the average log2 values of all three biological replicas for each subcellular compartment.

To compare the abundance of proteins assigned to specific compartments in T and PM fraction, we performed pair-wise comparisons of iBAQ values using log2 ratio. For these comparisons, proteins not identified in one fraction (having zero iBAQ) were assigned 0.2 to allow inclusion in the calculations. For each compartment, the calculated mean values of log2 are shown in **Figure 4**. In both leaf and root tissue, the fold increase of PM protein was significantly higher than that of proteins in other compartments, and proteins in CY, PL, EX, PR and CS were decreased in PM fraction. In general, the representation differences detected in different locations is consistent with the abundance comparisons, and both results support the capability of the technique for enriching the PM proteins.

### 3.3 Analysis of PM proteins identified in PM fractions

#### 3.3.1 Analysis of transmembrane domain of PM proteins

**Figure 5:**
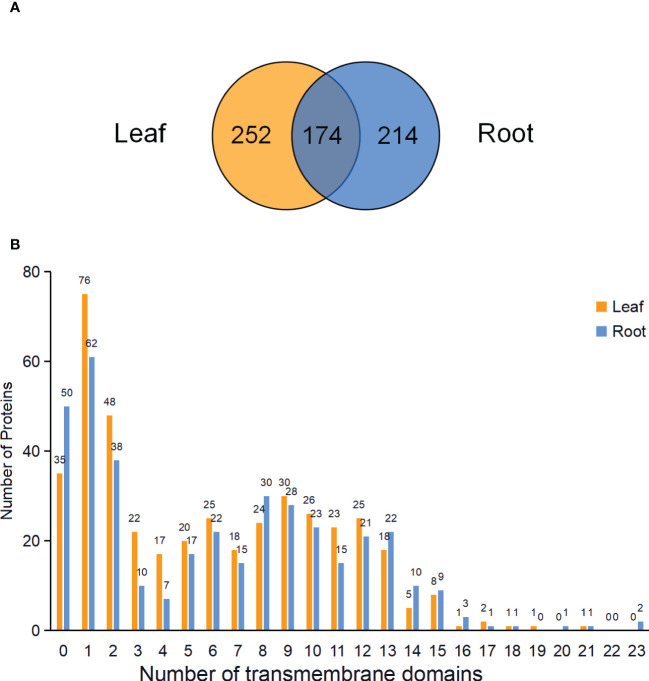
Venn diagram and analysis of transmembrane domains of identified PM proteins in leaves and roots. **(A)** Venn diagram showing the common and specific PM proteins in leaves and roots. **(B)** Predicted transmembrane domains of PM proteins in the leaves and roots.

In total, 426 and 388 proteins were predicted to be PM proteins in leaves and roots, respectively. Among identified PM proteins, 174 proteins were shared by leaves and roots, whereas 252 and 214 proteins were leaf- and root-specific. (**Figure 5A**; **Table S3**). To investigate the presence of transmembrane domain in these identified PM proteins, the analysis was performed using the TMHMM prediction programs. The results showed that 91.78% of proteins in leaves and 87.11% of proteins in roots had at least one transmembrane domain, and these proteins could be integral proteins (**Figure 5B**). The proteins without transmembrane domain could be peripheral proteins or lipid-anchored proteins.

#### 3.3.2 Functional classification of PM proteins

**Figure 6:**
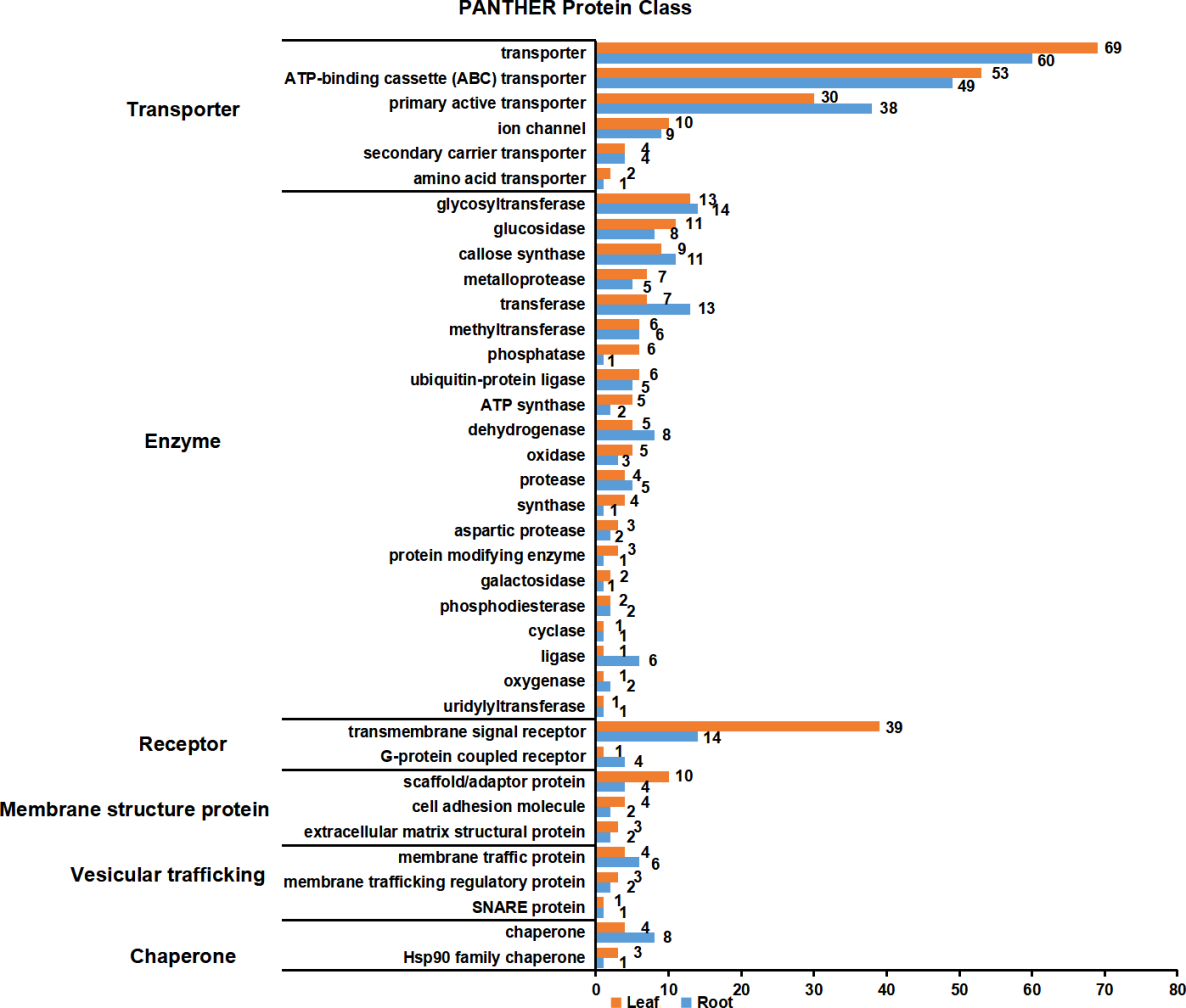
Functional classification of PM proteins in leaves and roots. The function of PM proteins are classified according to PANTHER classification system. The PM proteins were divided into six major categories: transporter, enzyme, receptor, membrane structure protein, vesicular trafficking and chaperone. Orange and blue blocks indicate the number of PM proteins in leaves and roots, respectively.

PM proteins are involved in many important biological processes. To investigate the functions of the identified PM proteins, functional classification has been performed using the PANTHER classification system (Mi et al., 2019). The results showed that the functions of proteins could be divided into six major categories: transporter, enzyme, receptor, membrane structure protein, vesicular trafficking and chaperone (**Figure 6**; **Table S4**). Transporter proteins dominated in the identified proteins, and ABC transporters constituted the largest group. A total of 53 and 49 ABC transporters were characterized in leaf and root, respectively, including members in ABCA, ABCB, ABCC and ABCG subfamily. Other major transporters included nitrate transporter (NRT1/2 ortholog), inorganic phosphate transporter (PHT1 ortholog), potassium transporter (POT4/5/7/11/12 ortholog), sulfate transporter (SULTR4.2 ortholog), metal-nicotianamine transporter (YSL6/7 ortholog), sucrose transporter (SUC2 and STP1 ortholog) and auxin transporter (PIN3 ortholog). Interestingly, nitrate and inorganic phosphate transporters were mainly identified from root PM proteome, while sucrose transporters were only identified from leaf. Primary active transporter was also the major category which included H^+^-ATPases, maganese-transporting ATPase, calcium-transporting ATPase and phospholipid-transporting ATPase. Enzyme proteins were the second largest class, in which glycosyltransferase contained the largest number. There were 13 and 14 glycosyltransferase proteins detected in the leaf and root PM proteome, respectively, and 23 of them were transmembrane proteins. A couple of important signal receptor proteins were characterized, including 18 Leucine-rich repeat (LRR) receptor protein kinases, 3 Lectin receptor kinases, 1 Cysteine-rich receptor-like protein kinases, 4 G-protein coupled receptors, Somatic embryogenesis receptor kinase 4 (SERK4) ortholog/BRASSINOSTEROID INSENSITIVE 1-associated receptor kinase 1 (BAK1) ortholog, Chitin elicitor receptor kinase 1 (CERK1) ortholog, Glutamate receptor 3.6 (GLR3.6) ortholog, and 15 other transmembrane signal receptors. Additionally, proteins involved in vesicular trafficking were also detected in the PM proteome, such as vesicle coat proteins (Clathrin heavy chain 1/2 ortholog) and SNARE proteins (Syntaxin-132 ortholog). In summary, the PM proteome has characterized the major type proteins localized on the PM, and many orthologs of functional PM proteins previously identified in other plant species have also been detected.

### 3.4 Identification and functional analysis of PM protein SgRKL1

#### 3.4.1 Identification and sequence analysis of PM protein SgRKL1

**Figure 7:**
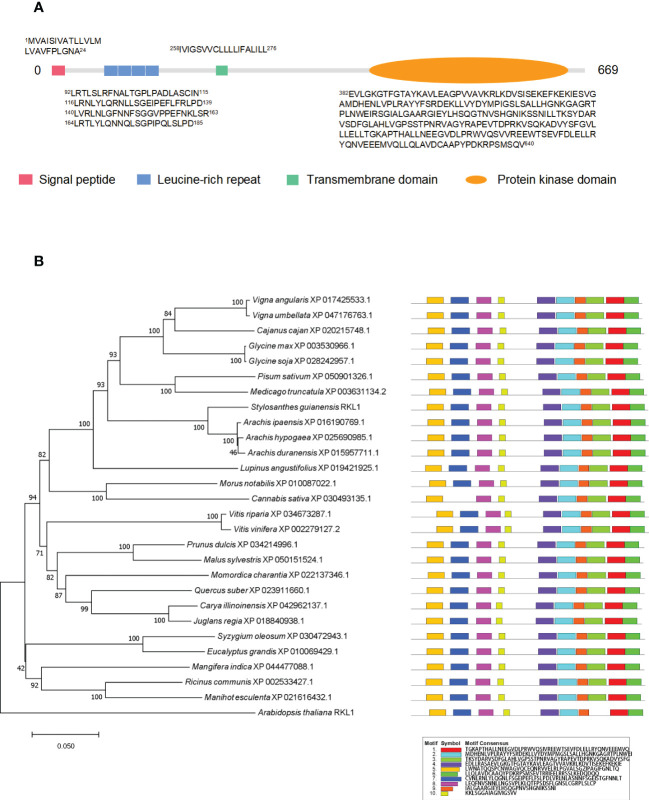
The domain, phylogenetic and motif analysis of SgRKL1. **(A)** The domain analysis of SgRKL1. The red box represents the signal peptide, blue box represents Leucine-rich repeat, green box represents the transmembrane domain, and the orange oval represents the protein kinase domain. The sequences of amino acid of each domain were listed correspondingly. **(B)** Phylogenetic tree and motif analysis of RKL1 protein sequences from 28 plant species. The legend represents the sequence of amino acids in the corresponding motifs.

In order to verify the PM localization of proteins identified from PM proteomics and also to investigate the potential involvement of PM proteins in biotic and abiotic stresses, we chose a newly identified receptor-like kinase, SgRKL1, for investigation. SgRKL1 was identified from both leaf and root PM proteomics of stylo, and its homolog in *Arabidopsis* was previously reported to regulate plant immunity and root growth (Tarutani et al., 2004; ten Hove et al., 2011; Zhao et al., 2019; Demirjian et al., 2022; Zhao et al., 2019). The sequence analysis showed that the coding sequence of SgRKL1 was 2010 bp in length, which encoded 669 amino acid residues (**Table S5**). The conserved domains analysis showed that SgRKL1 protein contained a signal peptide, 4 Leucine-rich repeats, a transmembrane domain and a protein kinase domain, belonging to the receptor-like kinases (RLKs) (**Figure 7A**). To investigate the conservation of RKL1 protein, phylogenetic analysis was performed using protein sequences of 28 plant species (**Table S6**). The results showed that RKL1 proteins from 26 species (except *Arabidopsis* and *Cannabis sativa*) contained 10 identical motifs, and *Arabidopsis* and *Cannabis sativa* also shared 9 common motifs with other plant species, indicating that RKL1 proteins were highly conserved across different species. SgRKL1 was clustered in the branch of leguminosa and had the highest homology with RKL1 of *Arachis ipaensis, Arachis hypogaea, Arachis duranensis* (**Figure 7B**).

#### 3.4.2 Validation of the subcellular localization of SgRKL1

**Figure 8:**
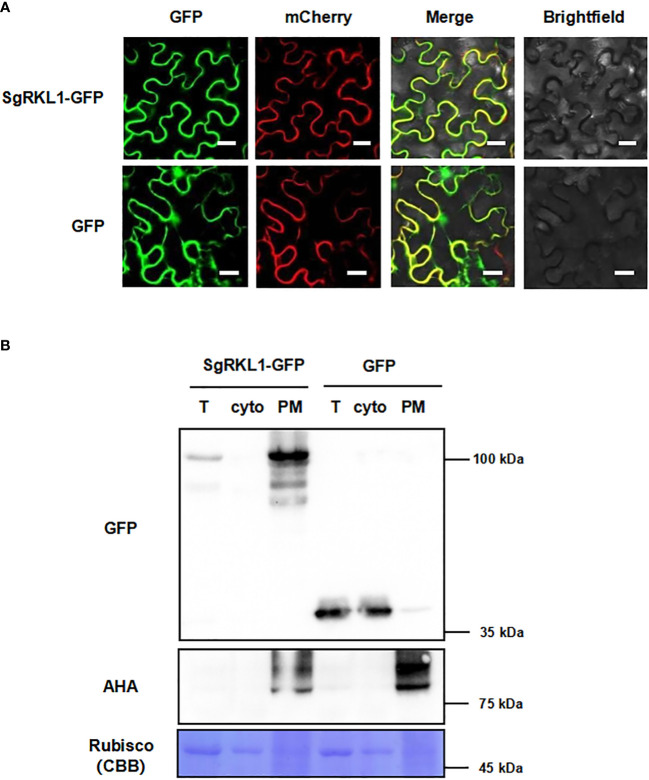
Subcellular localization of the identified PM protein SgRKL1. **(A)** Co-localization of SgRKL1-GFP and PM marker protein PIP2A-mCherry. Images were taken using confocal microscopy 2 days after infiltration in *N. benthamiana*. Bars, 20 µM. **(B)** Immunoblot analysis of SgRKL1-GFP transiently expressed in *N. benthamiana*. Total protein (T), cytosolic fraction (cyto), plasma membrane enriched fraction (PM) were collected 2 days after infiltration. Immunoblot analysis was performed using anti-GFP and anti-AHA antibodies to detect the distribution of SgRKL1-GFP/free GFP and PM marker protein AHA, respectively. Coomassie blue staining CBB was performed to show the distribution of Rubisco protein. The experiments were repeated three times with similar results as shown.

To verify the subcellular localization, we performed the co-localization and immunoblot analysis for SgRKL1. For co-localization analysis, the SgRKL1-GFP fusion protein and PM marker protein PIP2A-mCherry were transiently expressed in *N. benthamiana* leaves, and the fluorescent signals were observed using confocal microscopy. The results showed that the signal of SgRKL1-GFP was localized at PM and overlapped with PIP2A-mCherry, while the free GFP signal was mainly localized at cytoplasm and nuclues (**Figure 8A**). Meanwhile, the protein extracts of *N. benthamina* transiently expressed with SgRKL1-GFP and GFP were separated into total protein, cytosol and PM fraction using the same enrichment method, and the immunoblot analysis was performed to examine the localization of SgRKL1-GFP and GFP proteins. The results showed that the AHA proteins (PM marker) were significantly enriched in the PM fraction and depleted in the cytosol fraction, and the rubisco proteins (cytosolic marker) were depleted in the PM fraction, suggesting the successful separation of the protein extracts. SgRKL1-GFP proteins were detected in the total and PM fraction (significantly enriched in the PM fraction), while the GFP proteins were in the total and cytosol fraction, which was consistent with the results of co-localization analysis (**Figure 8B**). Both co-localization and immunoblot analysis have validated the PM localization of SgRKL1. These results also demonstrate that the enrichment method could be applied in the *N. benthamiana* system.

#### 3.4.3 Expression pattern analysis of *SgRKL1*

**Figure 9:**
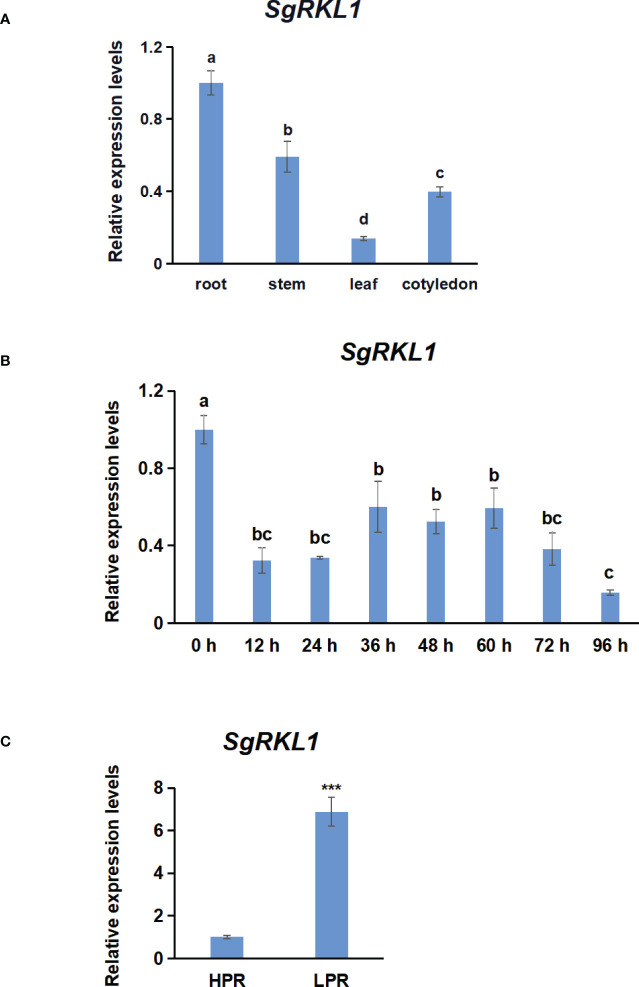
Analysis of expression pattern of *SgRKL1* by qPCR. **(A)** Expression pattern analysis of *SgRKL1* gene in different tissues of stylo under normal growth condition. **(B)** Expression pattern analysis of *SgRKL1* gene in leaves of stylo post inoculation with *C gloeosporioides*. Leaves samples were collected at 0 h,12 h,24 h, 36 h, 48 h, 60 h, 72 h and 96 h post inoculation of *C gloeosporioides*. **(C)** Expression pattern analysis of *SgRKL1* gene in roots of stylo under low phosphorus treatment. HPR represents the treatments with a phosphorus concentration of 300 μmol/L, and LPR represents the treatments with a phosphorus concentration of 0 μmol/L. Data are means ± SE pooled from three biological replicates, n = 3. Different letters in **(A)** and **(B)** indicate significant differences using Dancan’s test (*P* < 0.05). Asterisks in **(C)** indicate significant differences using the Student’s t-test (***P < 0.001).

To investigate the potential functions of *SgRKL1*, we performed the qPCR analysis to detect the expression patterns of *SgRKL1* in different tissue types of stylo (roots, stems, leaves, cotyledons) under normal growth condition and in response to biotic stress (inoculation with *C. gloeosporioides*) and abiotic stress (low phosphorus stress). The results showed that *SgRKL1* gene had highest expression in root and lowest expression in leaf (**Figure 9A**). The time-course expression pattern analysis of stylo post inoculation of *C. gloeosporioides* showed that the expression levels of *SgRKL1* in leaf were significantly down-regulated from 12 h to 96 h post inoculation (**Figure 9B**), whereas the expression levels of *SgRKL1* in root were up-regulated by the treatment of 15-day phosphorus starvation (**Figure 9C**). These results suggest that SgRKL1 may be involved in the responses against *C. gloeosporioides* infection and low phosphorus stress.

## 4 Discussion

Plasma membrane proteins play important roles in sensing signals and controlling solute transport. However, the identification of PM proteins is typically under-represented in the large-scale proteomics datasets because of the relatively low abundance and hydrophobicity of PM proteins (Yadeta et al., 2013). Plant PM proteins are even more challenging to isolate compared to those from mammalian cells due to the complexities and variation of plant species and tissue types. Improvement of PM protein identification could be achieved by removal of highly abundant soluble proteins (such as the RuBisCo large subunit) and other non-PM membrane proteins (Chen and Weckwerth, 2020). The method of isolating total microsomal membranes by differential centrifugation followed by treatment with nonionic detergent Brij-58 has greatly enriched PM proteins in cell culture and seedlings of *Arabidopsis* and root tissue of maize (Zhang and Peck, 2011; Zhang et al., 2013; Collins et al., 2017). Our results have also shown the successful application of such PM enrichment method in both leaf and root tissue of tropical forage stylo. For the immunoblot analysis of marker proteins in different fractions, the results in both leaf and root tissues of stylo have shown that the AHA proteins (PM marker) were strongly enriched in the PM fraction. The AOX1/2 proteins (mitochondrial marker) were enriched in the CM fraction in both leaf and root tissue of stylo but depleted in the PM fraction in leaf and decreased in the PM fraction in root. These results are consistent with those from the cell culture and seedlings of *Arabidopsis* and roots of maize (Zhang and Peck, 2011; Zhang et al., 2013; Collins et al., 2017). For the MS analysis of organellar composition in PM fractions, both our results in stylo and previous results in *Arabidopsis* have shown the increased representation of PM proteins in the PM fraction. Additionally, the PM fraction in stylo has also slightly increased the number of proteins from vacuole (VU), mitochondria (MI), golgis apparatus (GO) in leaf and VU in root, respectively. The number of proteins in cytosol (CY), plastid (PL), extracellular space (EX), peroxisome (PR), cytoskeleton (CS) has been decreased in PM fraction. In *Arabidopsis*, the PM fraction has enriched for proteins in CY and EX while decreased the number of proteins from VU, PL, MI and PR (Zhang and Peck, 2011). The difference in the organellar composition may be caused by different fraction comparison (PM versus total fraction in stylo whereas PM versus CM fraction in *Arabidopsis*) or different buoyancy of proteins in different plant species. The leaf tissue of stylo is rich in polysaccharides and polyphenols, which makes the protein extraction and enrichment process more complicated. The successful application in leaf and root tissues of stylo has further broaden the possible application of such enrichment method in tissues rich in polysaccharides and polyphenols, such as many tropical crops. In addition, we have also shown the method could successfully enrich the PM fraction in the leaf tissue of *N. benthamiana*. Therefore, it is suggested that this technology may be broadly applicable to PM protein studies in diverse plant species and tissue types.

Although the effect of this method for enriching PM proteins is conclusive, the mechanism by which the Brij-58 performs such role is not clear. The Brij-58 is also called polyethylene glycol hexadecyl ether with the chemical formula of HO(CH_2_CH_2_O)_20_C_16_H_33_. The Brij-58 may invert the PM vesicle inside-out and release other contaminants such as membrane organelles and/or cytosolic proteins (Zhang and Peck, 2011). It is also possible that Brij-58 selectively solubilizes other non-PM membrane proteins (Kaakinen et al., 2008). In addition, the enrichment method can not unequivocally assign the location of a protein to PM or isolate highly purified PM samples as two-phase partitioning method. However, it serves as a rapid, cheap and simple method to increase the representation of PM proteins in proteomic studies. Especially, the method is easier to handle when many samples need to be processed at the same time. Moreover, the enrichment for PM proteins will be sufficient to obtain meaningful information for many proteomic comparison. For instance, quantitative proteomic analysis of the PM-enriched protein fractions have identified the proteins with differential abundance in different growth zone of maize primary root and under water stress condition (Voothuluru et al., 2016).

In previous stylo proteomic studies, only 23 and 37 PM proteins have been detected in the leaf and root proteome, respectively (Liu et al., 2019). Our studies have identified 426 and 388 PM proteins in the enrichment fraction of leaf and root tissue, greatly increasing the identification of PM protein. In addition to PM protein purple acid phosphatases, many proteins that may play important biological functions in biotic and abiotic stress have been characterized. For instance, ABC transporters, nitrate transporter (NRT1/2 ortholog), inorganic phosphate transporter (PHT1 ortholog), potassium transporter (POT4/5/7/11/12 ortholog), sulfate transporter (SULTR4.2 ortholog), metal-nicotianamine transporter (YSL6/7 ortholog), sucrose transporter (SUC2 and STP1 ortholog), auxin transporter (PIN3 ortholog) and many primary active transporters have been characterized. As been demonstrated to drive the efflux or influx of a variety of substances including auxin, ABA, heavy metals and antimicrobial compounds in other plant species (Lane et al., 2016; Ye et al., 2021), these proteins may contribute to the adaptation of stylo to acidic and infertile soils. Therefore, it would be interesting to perform extensive proteomic analysis using the PM enrichment method to investigate resistance mechanisms of stylo against P deficiency as well as Al and Mn toxicity. In addition to transporters, many important receptor proteins that are essential in pathogen perception and resistance have also been characterized, such as LRR receptor protein kinases, Lectin receptor kinases, Cysteine-rich receptor-like protein kinases, G-protein coupled receptors, SERK4/BAK1 ortholog, CERK1 ortholog and GLR3.6 ortholog (Tang et al., 2017). These results also suggest the possible application of the PM enrichment method to study the stylo-pathogen interaction.

A newly identified receptor-like kinase, SgRKL1, has been characterized in both leaf and root PM proteome of stylo. To investigate the possible functions of SgRKL1, we have performed the sequence analysis, subcellular localization analysis and expression pattern analysis. The results have shown that SgRKL1 localizes on PM and may involve in regulating the responses against *C. gloeosporioides* and low phosphorus stress. Studies in *Arabidopsis* have also shown the role of *Arabidopsis RKL1 (AtRKL1)* in regulating plant immunity and root growth. For instance, the expression levels of *AtRKL1* in leaf are suppressed by the infection of *Pseudomonas syringae* pv. *Maculocola* (Tarutani et al., 2004). A genome-wide association analysis of two *Arabidopsis* mapping population has also identified AtRKL1 as the susceptibility factors targeted by *Ralstonia solanacearum*, and knocking out *AtRKL1* increases the resistance against *R. solanacearum* (Demirjian et al., 2022). Consistent with results in *Arabidopsis*, our results have also shown that the expression levels of *SgRKL1* decrease in response to the inoculation of *C. gloeosporioides*. All these results, together with high conservation of RKL1 proteins across different plant species, indicate that RKL1 proteins may be an important targets of pathogens and negatively regulate the plant immunity. In addition to the role in disease resistance, RKL1 may also be an important regulator in roots. In *Arabidopsis*, *AtRKL1* is expressed in vascular tissues throughout the root system, and *Atrkl1* mutants show reduced root length (ten Hove et al., 2011). Our studies also show that *SgRKL1* is highly expressed in root of stylo under normal growth conditions, suggesting the role of *SgRKL1* in regulating the root growth of stylo. The adjustment of root morphology is a common strategy in many plants to adapt to low phosphorus stress (Liu, 2021). Our studies show that the expression levels of *SgRKL1* in roots are significantly up-regulated by low phosphorus treatment, suggesting that *SgRKL1* may be involved in regulating the root adaptation to low phosphorus stress, but the mechanisms need further investigation.

## Data availability statement

The datasets presented in this study can be found in online repositories. The name of the repository and accession number can be found below: ProteomXchange; PXD037423.

## Author contributions

LY: Performed the experiments, analyzed the data, wrote the manuscript. JG: Performed the experiments, analyzed the data. MG: Performed the experiments. LJ: Conceived and designed the experiment, analyzed the data, wrote and revised the manuscript. LL: Revised the manuscript. All authors contributed to the article and approved the submitted version.
